# Nanoparticle albumin-bound paclitaxel versus solvent-based paclitaxel in breast cancer: A protocol for systemic review and meta-analysis

**DOI:** 10.1097/MD.0000000000024514

**Published:** 2021-02-19

**Authors:** Bingxue Li, Xinjie Chen, Tongjing Ding, Yihua Liu, Tingting Ma, Ganlin Zhang, Xiaomin Wang

**Affiliations:** a Beijing University of Chinese Medicine, Chaoyang District; b Department of Oncology, Beijing Hospital of Traditional Chinese Medicine, Capital Medical University, Dongcheng District, Beijing, China.

**Keywords:** breast cancer, efficacy, meta-analysis, nab-paclitaxel, paclitaxel, toxicity

## Abstract

**Background:**

Nanoparticle albumin-bound paclitaxel (nab-PTX) has exhibited clinical efficacy in breast cancer treatment, but toxicities can be yielded more at the same time. We did this meta-analysis aiming to unambiguously compare nab-PTX with conventional solvent-based paclitaxel in breast cancer patients of all stages.

**Method:**

Pubmed, EMBASE, Cochrane Library, Chinese Biomedical database, Chinese National Knowledge Infrastructure, Chinese Science and Technology Periodical database, and WangFang database were searched for head-to-head randomized controlled trials of nab-PTX and solvent-based paclitaxel in breast cancer. Other sources will also be searched like Google Scholar and gray literatures. Two researchers will independently search the database and extract data from the articles. Risk of bias will be assessed using the Cochrane Collaboration's tool. Objective tumor response rate, chemotherapy completion rate after 4 or 6 cycles, and toxicity will be primary outcomes. Disease control rate, overall survival, and progression-free survival/disease-free survival will be included in secondary outcomes. Risk ratio with 95% confidence interval was used for dichotomous variables while hazard ratio was used for time-to-event outcomes. The following 3 data sets will all be considered when synthesizing the data: intention-to-treat population, those who actually received taxanes treatment, and those who were actually assessed. All the analyses were done using Review Manager Software 5.3. Any disagreements in study selection, data collection, and analysis will be resolved by a third investigator.

**Results and conclusion:**

This study is aim to evaluate the efficacy and safety of nab-PTX compared with PTX in breast cancer treatment as well as to find the best dose or schedule and identify the benefit population. This meta-analysis could provide evidence for clinicians to make a better choice between nab-PTX and PTX in different specific contexts.

**Prospero registration number:**

CRD42019117912.

## 1 Introduction

The incidence and mortality of breast cancer, which are respectively 46.3 per 100,000 and 13 per 100,000, rank first in women cancer patients worldwide, according to the data of GLOBOCAN 2018.^[1]^ Nearly 36% of the females firstly diagnosed with breast cancer already have reginal or distant metastasis and 89.9% people survive 5 years or more after being diagnosed with female breast cancer.^[2]^ Except for stage I or part of stage II estrogen receptor positive/human epidermal growth factor receptor 2 negative breast cancer patients, nearly all breast cancer patients should undergo chemotherapy to gain a better prognosis.^[3]^

Among the most widely used chemotherapy agents taxanes stand out in the treatment of both early-stage and metastatic breast cancer.^[3,4]^ The term “taxanes” describes a group of drugs of similar structures and they work by blocking the microtubules from breaking down and thus inhibiting proliferating cells (including cancer cells) by forcing them to arrest in G2/M phase. The first drug of this kind developed is paclitaxel (solvent-based paclitaxel [sb-PTX]). It was first identified and isolated from Pacific yew tree (*Taxus brevifolia*, native to western North America) in 1971 as part of a National Cancer Institute program screening medicinal plants for potential anticancer activity^[5]^ and has been administrated to treat breast cancer by FDA since 1994. Hypersensitivity reactions may occur in 30% to 40% of patients using sb-PTX caused by sb-PTX itself or the solvent Cremophor EL. And thus dexamethasone and H1 and H2 -receptor antagonists should be used as premedication to avoid hypersensitivity reactions.^[6]^ Nanoparticle albumin-bound paclitaxel (nab-paclitaxel [nab-PTX]), a solvent free nanometersized form of paclitaxel, was initially invented in 1992 to avoid the toxicities associated with castor oil as it can be administered with shorter fusion schedule (30 minutes) and no premedication^[7,8]^ and it has been administrated to treat breast cancer by FDA since 2005. Nab-PTX includes 6 or 7 PTX molecules bound noncovalently^[9]^ to an albumin molecule forming a PTX-albumin primary aggregate of 4 to 14 nm.^[10]^ These then further aggregate to form an albumin-PTX particle of approximately 130 nm in diameter.^[11]^ Unlike sb-PTX entrapped in solvent micelles, nab-PTX outperforms it in drug distribution, clearance, systemic exposure as well as transportation to tumors and tumor uptake of PTX.^[11]^ Compared with sb-PTX, nab-PTX had demonstrated a higher objective response rate (33% vs 19%, *P* = .001) and a longer time to tumor progression (23.0 vs 16.9 weeks, hazard ratio (HR) = 0.75, *P* = .006) in metastatic breast cancer patients.^[7]^ This efficacy advantage can be explained by breaking through the limitation of drug solvent. Numbers of other clinical studies^[12–16]^ have also conformed the efficacy of nab-PTX. However, along with the higher drug accumulation in breast tumor, PTX-related toxicities (such as hematologic adverse events and sensory neuropathy) occurs more frequently at the same time.^[7,17]^

Despite of the appreciation of nab-PTX, data of head-to-head comparisons of nab-PTX and sb-PTX in breast cancer of all stages have not been fully explored. What's more, the completion rate of the planned cycles as well as subgroup analyses related to dose schedule and molecular subtype. We performed this meta-analysis of all the head-to-head randomized controlled trials of nab-PTX and sb-PTX in breast cancer to compare nab-PTX and sb-PTX in breast cancer patients comprehensively and objectively.

## 2 Methods

### 2.1 Study registration

This protocol will be prepared according to recommendations of the preferred reporting items for systematic review and meta-analysis protocols. It was registered on PROSPERO (CRD42019117912).

### 2.2 Search methods for study identification

Pubmed, EMBASE, Cochrane Library, Chinese Biomedical database, Chinese National Knowledge Infrastructure, Chinese Science and Technology Periodical database, and WangFang database will be searched for relevant studies published before December 31, 2020. The major search terms are (“breast neoplasms” or “breast cancer,” or “breast tumors”) and (“albumin-bound paclitaxel” or “Abraxane” or “ABI007”) and (“paclitaxel” or “Taxol” or “NSC125973”). We will also search Google Scholar or other gray literatures through the websites of American Society of Clinical Oncology, Chinese Society of Clinical Oncology, and European Society for Medical Oncology. Detailed search methods in Pubmed, EMBASE, and Cochrane Library could be get in Supplemental Digital Content.

### 2.3 Criteria for study selection

#### 2.3.1 Types of studies

Only randomized clinical trials about taxanes in breast cancer will be included in this study. Observational studies, case reports, and animal studies will be excluded.

#### 2.3.2 Types of participants

This study will consider patients at any age with histologically confirmed breast cancer regardless of clinical stage. However, those with unclear data on outcomes will be excluded.

#### 2.3.3 Types of interventions

The intervention in eligible studies is restricted to nab-PTX compared with PTX. Studies with more than 2 arms but include these 2 groups will also be included into analysis. But those comparing docetaxel with PTX or nab-PTX will be excluded.

#### 2.3.4 Types of outcome measures

##### 2.3.4.1 Major outcomes

- Objective tumor response rate (ORR) the proportion of participants with a complete or partial response.
- Chemotherapy completion rate (CCR) the proportion of those who complete taxanes treatment for 4 cycles or 6 cycles in all the patients.
- Toxicity as defined by the original study. Criteria might vary slightly but it will be noted in the result part. Adverse event related discontinuation will also be analyzed.

##### 2.3.4.2 Secondary outcomes

- Disease control rate (DCR) the proportion of participants with a complete response, partial response or stable disease.
- Overall survival (OS) time from date randomized to date of death (any cause).
- Progression-free survival (PFS)/disease-free survival (DFS) the time from date randomized to date of progression or death (any cause).

### 2.4 Data collection and analysis

#### 2.4.1 Selection of studies

**Figure 1:**
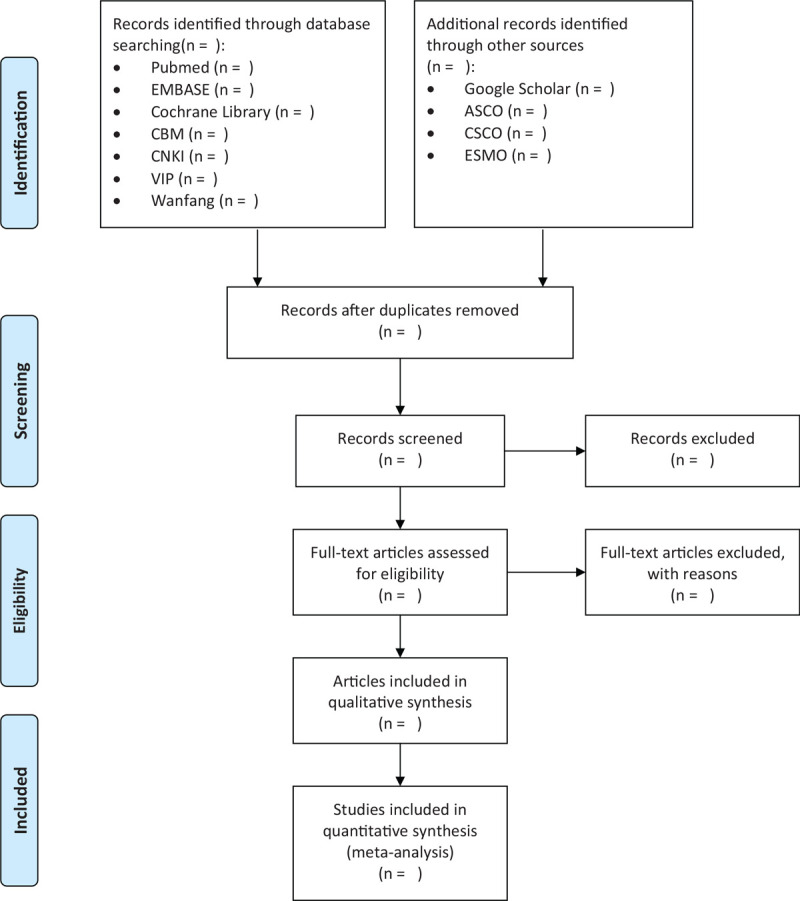
Flow diagram of this study selection. ASCO = American Society of Clinical Oncology, CBM = Chinese Biomedical database, CNKI = China National Knowledge Infrastructure, CSCO = Chinese Society of Clinical Oncology, ESMO = European Society for Medical Oncology, VIP = Chinese Science and Technology Journal Database.

Randomized controlled trials of patients at any age with histologically confirmed breast cancer which compares nab-PTX with sb-PTX with full publications or abstract will be included. Studies only available as protocols and studies published without outcomes of interest in this review will be excluded. The selection procedure will be conducted by 2 investigators independently. Any disagreements will be resolved through a consensus with the attendance of a third investigator. The selection process of eligible papers is shown in a preferred reporting items for systematic review and meta-analysis flow diagram (Fig. 1).

#### 2.4.2 Assessment of risk of bias

The Cochrane Collaboration's “Risk of bias” assessment tool will be used to assess the potential sources of bias in the included studies. This quality assessment will be based on random sequence generation (selection bias), allocation concealment (selection bias), blinding of participants and personnel (performance bias), blinding of outcome assessment (detection bias), incomplete outcome data (attribution bias), selective reporting (reporting bias), and others. Detection bias was also grouped by outcomes with similar risks of bias:

(1) ORR, DCR, PFS/DFS;
(2) CCR, OS, hematological toxicity;
(3) Nonhematological toxicity.

Each domain will be graded as “high,” “low,” or “unclear” following the criteria outlined in the Cochrane Handbook for Systemic Reviews of Interventions.^[18]^ For attribution bias, if the censor method is not clear in time-to-survival analysis, it should also be graded as at high risk. Two personnel will do the assessment independently and the result of other meta-analyses with published risk of bias summary will also be referred to. The final evaluation result will be got on the basis of the 4 outcomes mentioned above and if there were still unsolvable conflicts, a third person will be asked to give the final judgement.

#### 2.4.3 Data extraction and management

We will extract data on study randomization methods, participants’ baseline characteristics (age, gender, TNM stage, molecular subtype, etc), chemotherapy regimens (dose and schedule), outcomes together with outcome definitions both in printed papers, and electronic files (excel).

For ORR and DCR, numbers of patients in the following 3 data sets will also be specially extracted: intention-to-treat (ITT) population, those who actually received taxanes treatment, and those who were actually assessed (summing patients of complete release, partial release, stable disease, and progression disease) for tumor response. And in neoadjuvant treatment, we will only extract tumor response rate before surgery other than pathological complete response rate to evaluate the pure effect of chemotherapy and prevent the influence of other factors such as surgery. As for CCR assessment, the former 2 data sets will be used. And since time-to-event analysis including OS and PFS/DFS are usually done in ITT population in the original paper, analysis of them will only be done in ITT population and no considerations will be taken towards the choice of data sets.

For toxicity, we will extract numbers of grade 3/4 events for all the side effects reported in the papers. But only those reported by more than 2 studies will be included in the quantitative analysis. If grade 3/4 nausea and vomiting are reported separately, we will use data for vomiting. The denominator of toxicity will be the number of those who actually received the treatment at least 1 dosage.

Two investigators will independently extract the data and resolve queries through discussion with a third one. For trials with 3 arms including nab-PTX and sb-PTX, we will only extract data related to nab-PTX and sb-PTX. When there are more than 1 publication on the same study, we will use the updated data for the long-time follow-up outcomes.

#### 2.4.4 Measures of the treatment effect

Treatment outcomes of extracted available outcome data will be meta-analyzed. We will analyze ORR, CCR, DCR, and toxicity as dichotomous variables and derive a pooled risk ratio (RR) with 95% confidence interval. HR will be used to analyze time-to-event outcomes (including OS, PFS/DFS). The HR and associated variances will be extracted from the publications directly when possible. If it is not reported, we will use Engauge Digitizer to obtain the survival rate at different time points from Kaplan–Meier curves and calculated HRs and SEs using the spreadsheet shared by Jayne F Tierney.^[19]^

#### 2.4.5 Dealing with missing data

As for unclear or missing data, attempts will be made to contact the original investigators via E-mail. But if further detail is not available, data will be excluded when we conduct the analysis. We will also discuss the possible influence of the missing data on the outcomes in the review at the same time.

#### 2.4.6 Data synthesis

For ORR, CCR, DCR, OS, PFS/DFS, fixed-effect model (Mantel–Haenszel analysis) will be used at first when perform the analysis but if apparent clinical heterogeneity is found, then we will change into the random-effect model. However, for toxicity, random-effect model (Mantel–Haenszel analysis) will be used since the potential inevitable heterogeneity exists among different patients. Forest plots will be used to intuitively show the statistical difference.

For ORR, CCR, DCR, RRs larger than 1.0 favor nab-PTX. For OS, PFS/DFS, and toxicity, HRs or RRs less than 1.0 favor nab-PTX.

All the analyses will be done using Review Manager Software 5.3.

#### 2.4.7 Assessment of heterogeneity

The Chi^2^, the *I*^2^, and visual inspection of forest plots will be used to test for heterogeneity over all trials. A *P*-value of .05 is used to determine statistical significance. The interpretation of the *I*^2^ is as follows:

(1) 0% to 40%: might not be important.
(2) 30% to 60%: may represent moderate heterogeneity.
(3) 50% to 90%: may represent substantial heterogeneity.
(4) 75% to 100%: considerable heterogeneity.

#### 2.4.8 Assessment of reporting bias

If more than 10 studies are included in the meta-analysis, funnel plots and Egger test will be used to assess the reporting bias or small study effects. Publication might exist when the probability of the Egger test is <10%.

#### 2.4.9 Subgroup analysis

The following subgroup analyses will be done:

- treatment setting (neoadjuvant chemotherapy, adjuvant chemotherapy; metastatic chemotherapy);
- taxanes naïve or not;
- dose density (weekly or dose dense regimens);
- dose intensity (this was graded according to the dose intensity of nab-PTX: 120 to 130 mg/m^2^ per week as grade 1, 110 to 120 mg/m^2^ per week as grade 2, 90 to 110 mg/m^2^ per week as grade 3, 70 to 90 mg/m^2^ per week as grade 4);
- molecular subtype.

Chi^2^ test for interaction was used to these subgroup analyses.

#### 2.4.10 Sensitivity analysis

We conducted the sensitivity analysis in 2 ways:

(1) Exclude any of the study.
(2) Change the effect model to verify the result synthesized.

Special attention will be paid towards sample size, the outcome of missing data, and methodological quality. This was done using Review Manager Software 5.3.

#### 2.4.11 Certainty assessment

Grades of recommendation, assessment, development, and evaluation system will be used to assess the quality of the evidence. Two investigator will do the assessment independently and give a summary of finding table together. A third person will be necessary when there is any disagreement between the 2 investigators.

#### 2.4.12 Ethics and dissemination

Since this is a study on secondary analysis of the published articles and thus ethical approval is not required. The results will be published in a peer-reviewed journal and be presented at a relevant conference.

## 3 Discussion

Nab-PTX and sb-PTX are 2 frequently used medicine in chemotherapy of breast cancer. Previous studies^[20,21]^ have demonstrated that nab-PTX showed no inferiority in survival and priority in tumor response compared with other taxanes. But since they mix sb-PTX up with the other taxanes (especially docetaxel), the effect of dosage form has been disturbed by molecular structure. So we tend to analyze the data of only the nab-PTX and sb-PTX other than docetaxel or other taxanes.

To guarantee enough studies, we will include studies of breast cancer patients of all stages. And we will do subgroup analysis of different treatment regimens to minimize the heterogeneity caused by it. What's more, we will analyze the chemotherapy completion rate after 4 or 6 cycles and AE-related discontinuation to see the actual compliance of patients using nab-PTX regardless of the statistical significance of analysis in the original study. And for risk of bias assessment, we grouped the detection bias into 3 groups according to potential risk of bias and also censoring method (which is usually ignored in previous meta-analyses in cancer treatment) will be take into consideration and these strategies can make our meta-analysis more precise.

However, the number of studies to be included may be small. But we have included breast cancer patients of different stages and this may enlarge the sample size to some extent.

All in all, our meta-analysis will give us a comprehensive insight on the efficacy and toxicity of nab-PTX compared with sb-PTX in breast cancer patients and can help make clinical decision when choosing between the 2 drugs. Subgroup analysis will help to choose the most effective schedule and identify patients who might benefit more form nab-PTX.

## Author contributions

**Conceptualization:** Bingxue Li.

**Funding acquisition:** Ganlin Zhang, XiaoMin Wang.

**Investigation:** Bingxue Li.

**Methodology:** Xinjie Chen, Tongjing Ding, Yihua Liu.

**Writing – original draft:** Bingxue Li.

**Writing – review & editing:** Tingting Ma, Ganlin Zhang, XiaoMin Wang.

## Supplementary Material

Supplemental Digital Content
