# Supplementary material for: Nanoparticle albumin-bound paclitaxel versus solvent-based paclitaxel in breast cancer: A protocol for systemic review and meta-analysis
Source: Medicine (Baltimore). 2021 Feb 19;100(7):e24514. doi: 10.1097/MD.0000000000024514 (PMC7899816; doi:10.1097/MD.0000000000024514)
Supplement: Supplemental Digital Content [file medi-100-e24514-s001.docx]

## 1. PUBMED search strategy

("Breast Neoplasms"[Mesh] OR (Breast Neoplasm[tiab]) OR (Neoplasm, Breast[tiab]) OR (Breast Tumors[tiab]) OR (Breast Tumor[tiab]) OR (Tumor, Breast[tiab]) OR (Tumors, Breast[tiab]) OR (Neoplasms, Breast[tiab]) OR (Breast Carcinoma[tiab]) OR (Breast Carcinomas[tiab]) OR (Carcinoma, Breast[tiab]) OR (Carcinomas, Breast[tiab]) OR (Mammary Neoplasms, Human[tiab]) OR (Human Mammary Neoplasm[tiab]) OR (Human Mammary Neoplasms[tiab]) OR (Neoplasm, Human Mammary[tiab]) OR (Neoplasms, Human Mammary[tiab]) OR (Mammary Neoplasm, Human[tiab]) OR (Breast Cancer[tiab]) OR (Cancer, Breast[tiab]) OR (Mammary Cancer[tiab]) OR (Cancer, Mammary[tiab]) OR (Cancers, Mammary[tiab]) OR (Mammary Cancers[tiab]) OR (Malignant Neoplasm of Breast[tiab]) OR (Breast Malignant Neoplasm[tiab]) OR (Breast Malignant Neoplasms[tiab]) OR (Malignant Tumor of Breast[tiab]) OR (Breast Malignant[tiab]) OR (Breast Malignant Tumors[tiab]) OR (Cancer of Breast[tiab]) OR (Cancer of the Breast[tiab])) AND ("Albumin-Bound Paclitaxel"[Mesh] OR (Albumin Bound Paclitaxel) OR (Paclitaxel, Albumin-Bound) OR (Protein-Bound Paclitaxel) OR (Paclitaxel, Protein-Bound) OR (Protein Bound Paclitaxel) OR Abraxane OR (ABI007) OR (ABI-007) OR (ABI 007)) AND ("Paclitaxel"[Mesh] OR Anzatax OR (NSC-125973) OR (NSC 125973) OR (NSC125973) OR Taxol OR (Taxol A) OR (Bris Taxol) OR (Taxol, Bris) OR (Paclitaxel, (4 alpha)-Isomer) OR (Paxene) OR (Praxel) OR (7-epi-Taxol) OR (7 epi Taxol) OR (Onxol)) AND ((randomized controlled trial[pt] OR controlled clinical trial[pt] OR randomized[tiab] OR placebo[tiab] OR clinical trials as topic[mesh:noexp] OR randomly[tiab] OR trial[ti] NOT (animals[mh] NOT humans [mh])))

## 2. EMBASE (via EMBASE.com) search strategy

(‘breast cancer'/exp OR ‘Breast Neoplasm’:ab,ti OR ‘Neoplasm, Breast’:ab,ti OR ‘Breast Tumors’:ab,ti OR ‘Breast Tumor’:ab,ti OR ‘Tumor, Breast’:ab,ti OR ‘Tumors, Breast’:ab,ti OR ‘Neoplasms, Breast’:ab,ti OR ‘Breast Carcinoma’:ab,ti OR ‘Breast Carcinomas’:ab,ti OR ‘Carcinoma, Breast’:ab,ti OR ‘Carcinomas, Breast’:ab,ti OR ‘Mammary Neoplasms, Human’:ab,ti OR ‘Human Mammary Neoplasm’:ab,ti OR ‘Human Mammary Neoplasms’ ab,ti OR ‘Neoplasm, Human Mammary’:ab,ti OR ‘Neoplasms, Human Mammary’:ab,ti OR ‘Mammary Neoplasm, Human’:ab,ti OR ‘Breast Cancer’:ab,ti OR ‘Cancer, Breast’:ab,ti OR ‘Mammary Cancer’:ab,ti OR ‘Cancer, Mammary’:ab,ti OR ‘Cancers, Mammary’:ab,ti OR ‘Mammary Cancers’:ab,ti OR ‘Malignant Neoplasm of Breast’:ab,ti OR ‘Breast Malignant Neoplasm’:ab,ti OR ‘Breast Malignant Neoplasms’:ab,ti OR ‘Malignant Tumor of Breast’:ab,ti OR ‘Breast Malignant’:ab,ti OR ‘Breast Malignant Tumors’:ab,ti OR ‘Cancer of Breast’:ab,ti OR ‘Cancer of the Breast’:ab,ti) AND (‘Albumin Bound Paclitaxel’ OR ‘Paclitaxel, Albumin-Bound’ OR ‘Protein-Bound Paclitaxel’ OR ‘Paclitaxel, Protein-Bound’ OR ‘Protein Bound Paclitaxel’ OR Abraxane OR ‘ABI007’ OR ‘ABI-007’ OR ‘ABI 007’) AND (‘paclitaxel'/exp OR Anzatax OR ‘NSC-125973’ OR ‘NSC 125973’ OR ‘NSC125973’ OR Taxol OR ‘Taxol A’ OR ‘Bris Taxol’ OR ‘Taxol, Bris’ OR ‘Paclitaxel, (4 alpha)-Isomer’ OR ‘Paxene’ OR ‘Praxel’ OR ‘7-epi-Taxol’ OR ‘7 epi Taxol’ OR ‘Onxol’) AND (random*:ab,ti OR placebo*:de,ab,ti OR (double NEXT/1 blind*):ab,ti)

## 3. Cochrane search strategy

#1 MeSH descriptor: [Breast Neoplasms] explode all trees

#2 (breast):ti,ab,kw (Word variations have been searched)

#3 (neoplas*):ti,ab,kw (Word variations have been searched)

#4 (carcinoma*):ti,ab,kw (Word variations have been searched)

#5 (cancer):ti,ab,kw (Word variations have been searched)

#6 #3 OR #4 OR #5

#7 #2 AND #6

#8 #1 OR #7

#9 Albumin Bound Paclitaxel

#10 Paclitaxel

#11 #9 AND #10

#12 #8 AND #11
